# Effects of Air Frying on French Fries: The Indication Role of Physicochemical Properties on the Formation of Maillard Hazards, and the Changes of Starch Digestibility

**DOI:** 10.3389/fnut.2022.889901

**Published:** 2022-04-27

**Authors:** Lu Dong, Cai-yi Qiu, Rui-can Wang, Yan Zhang, Jin Wang, Jing-min Liu, Hua-ning Yu, Shuo Wang

**Affiliations:** ^1^Tianjin Key Laboratory of Food Science and Health, School of Medicine, Nankai University, Tianjin, China; ^2^MideaGroup Guangdong Midea Kitchen Appliances Manufacturing Co., Ltd., Foshan, China

**Keywords:** air frying, French fries, Maillard hazards, physicochemical properties, starch digestibility

## Abstract

This study focused on the formation of Maillard hazards in air fried fries, highlighting the correlation between the resultant physical properties of the fries and the formation of Maillard hazards. In the meantime, the effects of air frying on the *in vitro* starch digestibility of fries were explored. Potato strips were fried at various temperatures (180–200°C) and time (12–24 min). Results indicated that the extent of browning, hardness, and the contents of Maillard hazards (acrylamide, 5-hydroxymethylfurfural, methylglyoxal, and glyoxal) all increased steadily with air frying temperature and time. Moisture content were negatively correlated (*p* < 0.001) with Maillard hazards content and physicochemical properties except for L^*^ with the correlation coefficients range from −0.53 to 0.94, and positively correlated with L^*^ value with correlation coefficient was 0.91, hence, reducing the Maillard hazard exposure while maintaining the desired product quality can be achieved by controlling the moisture content of the air fried French fries. Compared with deep frying (180°C−6 min), air frying decreased acrylamide and 5-hydroxymethylfurfural content with the maximum reduction rate were 47.31 and 57.04%, respectively. In addition, the *in vitro* digestion results suggested that air frying resulted in higher levels of slowly digestible starch (48.54–58.42%) and lower levels of resistant starch (20.08–29.34%) as compared to those from deep frying (45.59 ± 4.89 and 35.22 ± 0.65%, respectively), which might contribute to more balanced blood sugar levels after consumption. Based on the above results, it was concluded that air frying can reduce the formation of food hazards and was relatively healthier.

## Introduction

Potatoes are one of the most consumed foods globally, and deep frying is the most common method of cooking (1). Deep frying gives rise to a variety of pigments and volatiles to fries through Maillard reaction, resulting in characteristic colors and aroma of fries. However, deep fried fries tend to have a higher oil content (about 40%), which is considered unhealthy due to potentially excessive fat intake (2). Moreover, it can lead to the production of a variety of Maillard hazards, such as acrylamide (AM), 5-hydroxymethylfurfural (5-HMF), heterocyclic amines, polycyclic aromatic hydrocarbon, and advanced glycation end products (3). In addition, some frying oils derived from plant seeds may contain lipophilic aflatoxin B_1_, and these aflatoxin B_1_ may be immersed into food along with the oil, which brings food safety hazards (4). French fries are the most widely acknowledge dietary source of AM (5), which has been classified as a probable carcinogen (Group 2A) by the International Agency for Research on Cancer (6, 7). In 2017, the European commission set a “benchmark level” for AM in French fries at 500 ng/g (8). 5-HMF is formed via Maillard reaction or caramelization reaction of hexose after hydrolysis. Moreover, it has been reported that 5-HMF can form AM by reacting with asparagine during heating (9). To date, the human health risks of 5-HMF are still controversial, but it can be metabolized into 5-sulfoxylmethylfurfural and 5-chloromethylfurfural in human body, which has potential genotoxicity and carcinogenicity (10). In addition, α-dicarbonyl compounds (αDCs) are important intermediates in Maillard reaction, such as glyoxal (GO), methylglyoxal (MGO), and 3-deoxyglucosone (3-DG), which arise from the degradation of Amadori compounds or the caramelization of sugars (11). They can react with the amino in dietary proteins, yielding advanced glycation end products, which associated with various chronic diseases, such as diabetes, aging and atherosclerosis (12, 13). For the sake of healthier foods, many attempts have been made to find inhibitors of the formation of Maillard hazards (14). For processed potatoes, it was suggested that using potato cultivars that are low in reducing sugars or asparagine, or treating potatoes with acids, flavonoids, or glutathione effectively reduced AM content in products (5, 15, 16). However, such pretreatments can cause deteriorated eating quality, limiting their practical application.

Air frying is an emerging processing technology that has been widely accepted by consumers. Instead of immersing food materials in oil, hot air is used as the heat transfer medium, by which it not only imparts comparably palatable products to deep frying products, but also shows health and environmental advantages, such as reduced oil consumption and zero effluent discharge (17–19). However, the physical properties of air fried food also have certain shortcomings. Gouyo et al. (20) reported that deep fried products are crispier than air fried ones because of more effective water loss that contributed to greater crispness and hardness. There have been studied the microstructure and sensory analysis in air fried fries (18), but information on the effects of air frying on the formation of Maillard hazards remains scare. Water is a key factor for fried food products (fries, chips, etc.). Moisture content not only determines the crispiness and hardness of the products, but also relates to the formation of Maillard hazards. Yuan et al. (21) reported the relationships between moisture content, lipid oxidation, and the production of AM and MGO, which suggested that extended frying and accelerated temperature led to reduced moisture content and intensified lipid oxidation. Considering the impacts of water on texture and the Maillard reaction, and that the latter plays a key role in color, there should be certain correlation between the physical (texture and color) and chemical (Maillard hazards) properties of the products. In other words, for a certain heat-processed food system like French fries, a predictive model could be established between sensory/physical properties and potential food hazards. Previous studies suggested that αDCs are involved in the subsequent formation of AM and 5-HMF. For example, in the fructose/asparagine model system, the correlation coefficient between MGO consumption and AM formation was 0.931 (22). It has found that the production of 5-HMF has a positive correlation with the content of 3-DG in wheat-flour biscuits (23). Nevertheless, the effect of αDCs on AM and 5-HMF formation in air frying process has not been fully studied.

Furthermore, potatoes contain 70–80% starch, which may potentially lead to high blood glucose levels after meals. Based on *in vitro* methods, starch can be divided into rapidly digestible starch (RDS), slowly digestible starch (SDS) and resistant starch (RS) (24). It has been reported that different cooking methods alter the microstructure and the nutritional value of food, and the microstructure might strongly link with the starch digestibility (25). RDS refers to the amount of starch hydrolyzed by α -amylase within 20 min, causing a rapid rise in blood sugar levels. SDS takes a long time (20–120 min) to digest and thus not making a great contribution to increase blood sugar. RS, unhydrolyzed after 120 min, is classified as dietary fiber, is not digestible, but can be fermented by bacteria in human colon to produce short-chain fatty acids, which plays an important role in preventing colon cancer, diverticulitis, and hemorrhoids (26, 27). Thus, *in vitro* starch digestibility provides an indicator of the glycemic index (GI) and potential health benefits/hazards of starchy foods. Cooked potatoes contain significant amounts of RS, and the digestibility of potatoes is related to cooking method, time, and temperature (28). However, the effects of air frying on the digestibility of potato starch by new technology had not been well-reported.

This study focused on the formation of Maillard hazards (AM, 5-HMF, αDCs) in air fried fries, highlighting the correlation between the resultant physicochemical properties (moisture content, texture, and color) of the fries and the formation of Maillard hazards. The effects of air frying on starch digestibility and RS content of French fries were also studied. Furthermore, based on the similar sensory score, the Maillard hazards, physicochemical properties, and starch digestibility of air fried French fried was compared with the deep fried French fried, which will provide a theoretical basis for the application of air frying technology in food processing.

## Materials and Methods

### Material and Reagents

AM, ^13^C-Acrylamide, 5-HMF, formic acid, methanol, and acetonitrile (HPLC grade) were obtained from Sigma-Aldrich (St. Louis, Mo., U.S.A.). 3-DG was supplied by Toronto Research Chemicals (Toronto, Canada). MGO (40% aqueous solution), GO (40% aqueous solution), *o*-phenylenediamine (OPA), *n*-hexane, potassium ferrocyanide trihydrate, zinc acetate dihydrate were purchased from Aladdin Bio-Chem Technology Co. Ltd. (Shanghai, China). Oasis HLB (6 mL, 200 mg) and Oasis MCX (3 mL, 60 mg) cartridges were supplied by Waters Corp. (Milford, MA, USA). French fries (9 × 9 mm) were obtained from Metro Chef ^®^ frozen French fries. Sunflower oil was bought from the local supermarket. Milli-Q System was used for preparing the ultrapure water (Millipore, St. Louis, MO. USA). Potassium hexacyanoferrate (3.6 g) and zinc acetate (7.2 g) were dissolved in 100 mL of water to prepared Carrez I and II solutions, respectively.

### Preparation of French Fries

Deep frying process was conducted in an electric fryer (HENG ZHI, GuangZhou, China) filled with 5 kg sunflower oil. Frozen French fries (300 g) were carefully poured into the oil when the center temperature reached 180°C and cooked for 6 min according to previous method (5). The center temperature of oil was measured with a TESTO 905T1 Probe Thermometer (Testo SE &Co. KGaA, Shanghai, China) with temperature measurement range is from −50 to 350°C.

Air frying was conducted in air fryer equipment (Toshiba ET-VD7250, Foshan, Guangdong, China). Frozen French fries (300 g) were put in the cooking basket, making sure that the stripes make little contact with each other. The air frying program was set for different temperatures (180, 190, 200°C) and times (12, 15, 18, 21, 24 min).

Three independent repetitions of each treatment condition were performed. All samples were cooled to room temperature. Each sample was ground to coarse powders for analyses except for texture analysis.

### Determination of Moisture Content

Moisture content was determined in an oven (BAO-80A, STIK instrument equipment Co., Ltd., Shanghai, China) heated at 102 ± 3°C until constant weight.

### Determination of Color Parameters

The color measurements of French fries were measured in a colorimeter (model CM-5; Konica Minolta Sensing, Inc., Osaka, Japan). L^*^ (lightness/darkness), a^*^ (greenness/redness), and b^*^ (blueness/yellowness) were used to express the color of French fries (29). The total color difference (ΔE) was calculated as follows:


$$\Delta E\;=\;\sqrt[{2}]{{\left(L^{*}-L\right)}^{2}+{\left(a^{*}-a\right)}^{2}+{\left(b^{*}-b\right)}^{2}}$$

where L, a, and b refer to the color values of the fresh potato strips.

### Determination of Texture

A TA-XT plus texture analyzer (Stable Micro Systems Co. Ltd., Surrey, UK) was used for the texture profile analysis (TPA) of French fries. French fries were cut into 9 × 9 × 20 mm cubes and placed on the center of the fixture with the rectangle size exposing to the probe. A P/36R probe was used to compress the sample, according to following settings: pre-test speed, 4.0 mm/s; test speed, 2.0 mm/s; post-test speed, 4.0 mm/s; Interval between two compressions, 5 s; strain: 40%; trigger force, 5 g. The textural parameters, hardness, and chewiness, were calculated from the TPA curve (30). Six cubes were used to measure the texture indices and the average reading of it was calculated for one independent experiment.

### Sensory Analysis

Ten postgraduates (age: 21–30, 5 males, 5 females) with food sensory evaluation experience were invited as panelists to score the samples in terms of color and texture, the score criteria of French fries were presented in Table 1. The panelists were trained according to national standard method (GB/T 16291) and were required to clean their mouth with water before the first taste and between samples.

**Table 1:** Score criteria on the sensory evaluation of French fries.

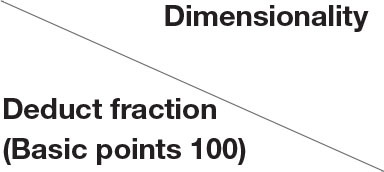	**Color**	**Texture**
0	<5% of the fries are black on both ends	Thin layer of brittle skin, soft inside, one end of the hand is straight and not bent
5	5–10% of the fries are black on both ends	More than 95% have a thin layer of brittle skin, soft inside, one end of the hand is straight and not bent
10	/	More than 90% have a thin layer of brittle skin, soft inside, one end of the hand is straight and not bent
Disqualification	Dark brown and white	Soft and burnt

### Determination of Maillard Hazards

#### Sample Pretreatment

A multiple stage extraction protocol was applied for pretreatment as described in Gökmen et al. (31). 1.00 g well-ground sample was weighted in a 50 mL centrifuge tube, and vortexed for 3 min in each step after extracting with 20 mL of water (10, 10 mL), then centrifugation at 8,000 × g for 10 min, and transfer the clear supernatant into a test tube. Then, 5 mL of *n*-hexane, 0.5 mL of Carrez I and 0.5 mL of Carrez II solutions were added to the supernatant, vortexed for 5 min, and centrifuged at 10,000 × g for 10 min to remove the fat and protein. This procedure was repeated twice. Subsequently, the resultant aqueous extract was used to analyze AM, 5-HMF, and αDCs.

#### Analysis of AM

The extract samples purified and quantitated protocols of AM refer to our previous report (32). Briefly, the extract was purified through an Oasis MCX column and filtered through a 0.22 μm filter membrane for analyze. Waters Acquity I Class UPLC System coupled to a TQ detector with electrospray ionization operated in positive mode was used for AM analysis. The product ions of AM were 54.8 and 44 with 8 V and 12 V collision energy; the product ions of ^13^C-acrylamide were 45 and 58 with 9 V collision energy. AM concentration in samples was quantified by internal standard calibration curve established in the range between 1.0 and 100 ng/mL spiked with 50 ng/mL ^13^C-acrylamide. The limit of detection (LOD) and the limit of the quantitation (LOQ) were 5.19, 17.33 ng/g, respectively. The recoveries ranged between 87.84 and 96.72%.

#### Analysis of 5-HMF

One milliliter of the extract was filtered through 0.22 μm filter membrane and analyzed by Waters Acquity I Class UPLC System in positive mode. Chromatographic separations were performed on an Symmertry C18 (4.6 × 150 mm, 5 μm) column at 30°C. The injection volume was 10 μL. 5% Methanol (A) and 95% (v/v) 0.1% formic acid (B) were used as the mobile phase (0–2 min, 5–20% A; 2–4 min, 20% A; 4–6 min, 20–5% A; 6–8 min, 5% A) at a flow rate of 0.5 mL/min. 5-HMF was eluted in 6.41 min. The electrospray source settings were the same as AM. The product ions of 5-HMF were 109 and 81 with 13 V and 17 V collision energy. The concentration of 5-HMF was quantified by the external calibration curve established in the range between 1 and 100 ng/mL. The LOD and LOQ were set at 1.26, 4.23 ng/g, respectively. The recoveries ranged between 73.82 and 80.44%.

#### Analysis of αDCs

Three milliliter of the extract was mixed with 100 μL of OPA solution (20 mg/mL) and shaken (100 rpm) for 20 min at 60°C and protected against exposure to light for the derivatization. Then the mixture was loaded onto the Oasis-HLB column (pre-balanced with 5 mL of methanol and 5 mL of water), and eluent was discharged. Next, 5 mL water was loaded and the eluent was discharged. Finally, 3 mL of the mixture of acetonitrile: water (1:1, v/v) was passed through the cartridge, the eluent was collected and filtered through 0.22 μm organic filter membrane and analyzed by Waters Acquity I Class UPLC System in positive ionization mode. Chromatographic separation was performed in Symmertry C18 (4.6 × 150 mm, 5 μm) at 40°C. 5% Methanol (A) and 95% (v/v) 0.1% formic acid (B) were used as the mobile phase (0–10 min, 30–60% A; 10–12 min, 60–30% A; 12–15 min, 30% A) at a flow rate of 0.4 mL/min. 3-DG, GO and MGO were eluted in 7.61, 10.76, and 12.49 min, respectively. The electrospray source settings were the same as AM. The fragment ions of m/z 235 → 199 (collision energy of 30 V), m/z 131 → 77 (collision energy of 11 V) and m/z 145 → 77 (collision energy of 14 V) were used to quantify quinoxaline derivatives of 3-DG, GO, and MGO, respectively. The levels of quinoxaline derivatives of 3-DG, GO and MGO were calculated using the external standard calibration curve established in the range between 1 and 100 ng/mL. The LOD of αDCs were 0.089–0.670 ng/g; LOQ were 0.297–2.235 ng/g; The recoveries ranged between 78.28 and 104.88%.

### *In vitro* Starch Digestibility

*In vitro* starch digestion method was conducted based on the protocol of Englyst et al. (1992) with slight modifications (33). The starch content of each fried sample was determined with the Megazyme Total Starch method. French fries containing 100 mg of starch were added to 4.0 mL of acetate buffer (0.2 M, pH 5.2) in a 50 mL centrifuge tube with 2 magnetic beads. Starch digestion was then initiated by adding 1.0 mL of the enzyme mixture, which was prepared by dissolving 0.738 g of α-Amylase in 12.0 mL of water, stirring at 37°C for 10 min, centrifuging at 5,000 × g for 10 min, then adding 50 μL of amyloglucosidase (3,300 U/mL) to the supernatant. The samples were digested at 37°C for 2 h under magnetic stirrer. During digestion, 50 μL aliquots were taken and mixed with 950 μL of absolute ethanol at 20, 40, 60, 80, 100, and 120 min, respectively. After that the samples were centrifuged at 13,000 × g for 5 min, the D-glucose (GOPOD Format) assay was used to determine the glucose content in the supernatant (34). The RDS, SDS, and RS amounts were calculated using the following equations:


$$\begin{array}{l} \;RDS\;=(G_{20}\;\times \;F\times 0.9\times 100)/TS\\ SDS\;=\;(G_{120}\;-G_{20})\;\times \;F\times 0.9\times 100/TS\\ \;RS\;=\;TS\;-\;(RDS\;+\;SDS) \end{array}$$

**G**_**20**_: Absorbance value for glucose at 20 min incubation.

**G**_**120**_: Absorbance value for glucose at 120 min incubation.

F: 100/GOPOD absorbance

TS: total starch weight (mg).

### Statistical Analysis

The data were drawn with Origin 8.0 software (OriginLab Corporation, Northampton, MA, USA) and expressed as mean ± standard deviation (SD). One-way ANOVA (*n* = 3 for all treatments) analysis was done using SPSS statistical software (Chicago, USA). Statistical significance was determined for *p*-value of < 0.05. The correlation between thermal processing hazards and sensory characteristics was evaluated by Pearson's correlation test of SPSS.

## Results

### The Physical Characteristics of Air Fried French Fries

The physical results (moisture content, color, texture, and sensory scores) of air fired French fries from different treatments are shown in Table 2. The moisture content decreased significantly with both the temperature and duration of the air frying treatment (*p* < 0.05). The L^*^, a^*^, b^*^ values of raw potatoes were 81.51 ± 0.27, −1.37 ± 0.15, and 28.44 ± 0.14, respectively. L^*^ decreased, while a^*^, b^*^, and ΔE increased with the air frying time and temperature. The texture attributes, including hardness and chewiness, showed the same trend as that of ΔE. The sensory score results indicated that air frying process at 180°C−21 min, 190°C−18 min, and 200°C−18 min obtained the best physical attributes.

**Table 2:** Changes in moisture content, color, texture, and sensory score of French fries during the production process.

**Samples**	**Moisture content**	L*	a*	b*	**ΔE**	**Hardness/g**	**Chewiness**	**Sensory score**
18°C 12 min	60.52 ± 0.07%°	76.45 ± 0.33^gh^	−0.39 ± 0.14^a^	32.21 ± 0.97^a^	6.46 ± 0.29^ab^	648.29 ± 121.88^a^	247.84 ± 17.53^a^	0
15 min	54.02 ± 0.36%^m^	76.14 ± 0.13^g^	0.65 ± 0.18^bc^	35.32 ± 0.55^bc^	8.96 ± 0.48^c^	783.42 ± 117.60^a^	306.86 ± 30.75^a^	91.5 ± 2.42
18 min	51.58 ± 0.19%^k^	74.95 ± 0.40^f^	1.35 ± 0.29^d^	36.49 ± 0.31^ce^	10.75 ± 0.06^d^	1029.42 ± 67.47^ab^	465.22 ± 71.23^bc^	92.5 ± 2.63
21 min	43.82 ± 0.16%^g^	71.97 ± 0.46^d^	3.07 ± 0.21^f^	38.14 ± 0.52^ef^	14.33 ± 0.10^gh^	1434.93 ± 47.87^c^	621.13 ± 109.18^de^	98 ± 2.58
24 min	35.11 ± 0.32%^d^	69.38 ± 0.97^b^	5.74 ± 0.20^h^	40.79 ± 0.37^g^	18.74 ± 0.35^j^	1950.58 ± 51.85^de^	736.70 ± 129.97^ef^	93 ± 2.58
190°C 12 min	59.22 ± 0.25%^n^	76.20 ± 0.20^g^	−0.02 ± 0.18^ab^	32.40 ± 0.85^a^	6.79 ± 0.58^b^	697.75 ± 219.68^a^	263.34 ± 18.52^a^	0
15 min	52.86 ± 0.39%^l^	75.68 ± 0.38^fg^	1.03 ± 0.30^cd^	36.70 ± 0.72^ce^	10.42 ± 0.29^d^	1031.41 ± 264.23^ab^	350.42 ± 45.63^ab^	92 ± 2.59
18 min	46.21 ± 0.08%^i^	73.60 ± 0.77^e^	2.62 ± 0.62^ef^	37.65 ± 1.54^df^	12.85 ± 1.17^ef^	1354.81 ± 105.82^bc^	553.31 ± 32.01^cd^	97.5 ± 2.64
21 min	40.87 ± 0.08%^e^	71.15 ± 0.26^cd^	4.62 ± 0.37^g^	38.70 ± 0.71^f^	15.78 ± 0.39^i^	1680.86 ± 78.07^cd^	720.57 ± 99.54^ef^	96 ± 3.94
24 min	32.98 ± 0.48%^b^	69.19 ± 0.64^b^	6.13 ± 0.37^h^	40.61 ± 1.56^g^	18.88 ± 1.55^j^	2606.59 ± 385.64^f^	1317.02 ± 6.57^h^	0
200°C 12 min	59.50 ± 0.21%^n^	77.39 ± 0.33^h^	0.02 ± 0.16^ab^	31.49 ± 0.63^a^	5.35 ± 0.42^a^	681.00 ± 80.66a	295.00 ± 13.60^a^	91 ± 2.11
15 min	49.92 ± 0.45%^j^	76.09 ± 0.24^g^	1.39 ± 0.43^d^	34.48 ± 0.26^b^	8.58 ± 0.33^c^	875.99 ± 39.76a	305.57 ± 35.56^a^	95 ± 4.08
18 min	45.19 ± 0.95%^h^	73.87 ± 0.25^e^	3.03 ± 0.45^f^	35.85 ± 0.48^bd^	11.52 ± 0.62^de^	1691.04 ± 55.27^cd^	629.37 ± 57.16^df^	97 ± 3.49
21 min	33.79 ± 0.27%^c^	70.60 ± 0.41^c^	4.39 ± 0.12^g^	36.88 ± 0.08^cf^	14.95 ± 0.31^hi^	2244.77 ± 194.28^ef^	960.82 ± 57.84^g^	95 ± 4.09
24 min	31.80 ± 0.11%^a^	64.70 ± 0.31^a^	7.79 ± 0.12^i^	41.96 ± 1.50^g^	23.46 ± 1.03^k^	4218.56 ± 389.62^g^	1399.97 ± 54.97^h^	0

*Different lowercase letters indicate significant differences (p < 0.05) between different production process*.

### AM, 5-HMF and αDCs Content in Air Fried French Fries

**Figure 1:**
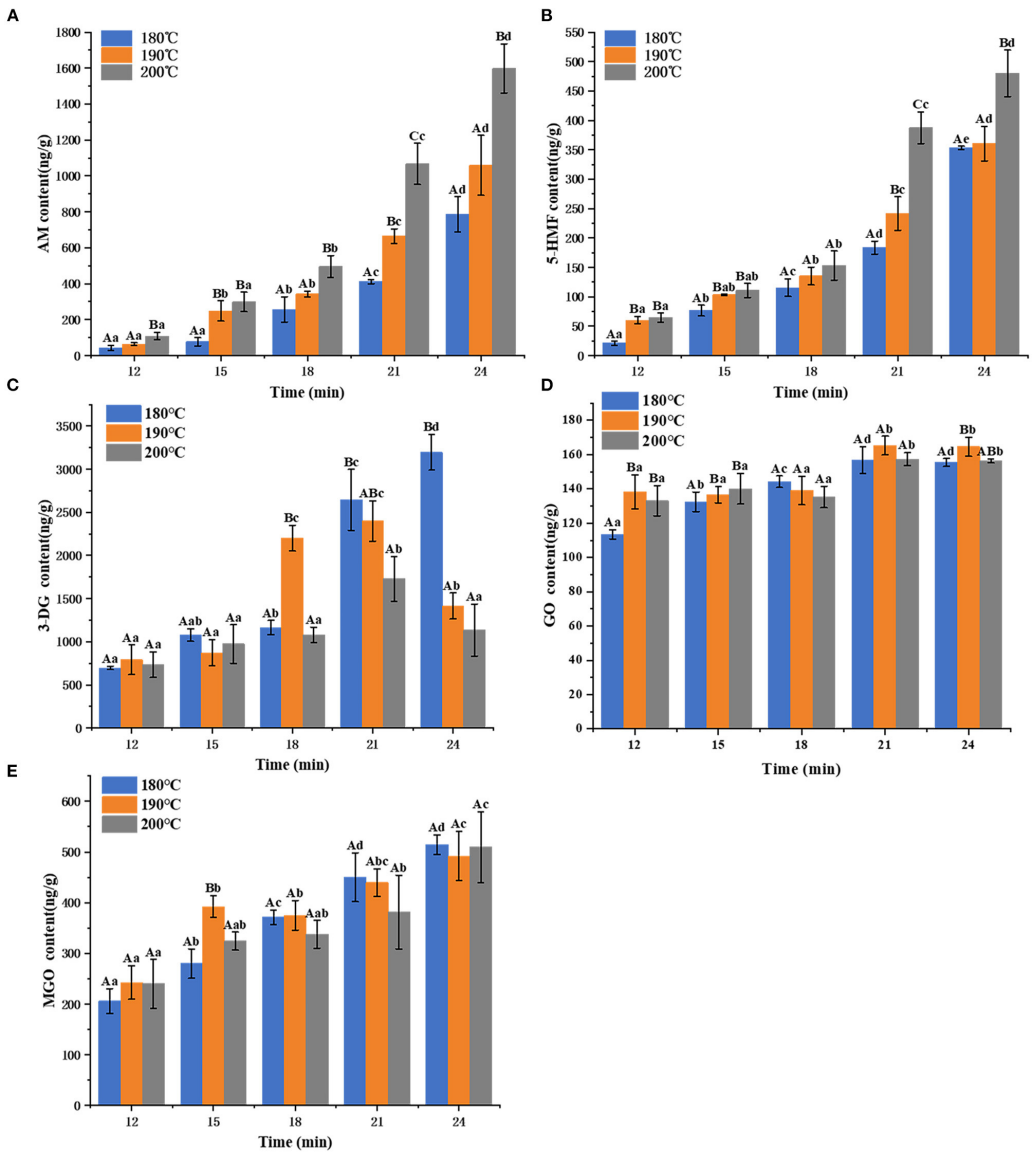
The content of AM **(A)**, 5-HMF **(B)**, 3-DG **(C)**, GO **(D)**, and MGO **(E)** in air fried fries under different time and temperature. Different capital letters **(A,B)** indicate significant (*p* < 0.05) difference of each sample among the temperature, and different lowercase letters (a,b) indicate significant (*p* < 0.05) difference among the samples at each time point.

The levels of Maillard hazards after the air frying processes are shown in Figure 1. The AM content of air fried French fries increased from 42.12 ± 14.46 ng/g to 1598.41 ± 136.62 ng/g as the increase of temperature and time. Likewise, the 5-HMF content of air fried French fries increased from 20.86 ± 3.55 to 479.88 ± 39.63 ng/g. For intermediate αDCs, the GO (form 113.42 ± 2.66 to 165.25 ± 5.45 ng/g) and MGO (from 205.81 ± 24.42 to 514.34 ± 19.29 ng/g) exhibited increasing trend with the time at the same air frying temperature. However, there was no significant difference in the content of GO and MGO in air fried French fries with different temperatures under the same time. The change of 3-DG (from 697.07 ± 18.44 to 3195.25 ± 207.48 ng/g) was not linear when the air frying temperature were 190 and 200°C, it exhibited increment before showing subsequent decline afterwards, whereas 3-DG content increased with the time when air fried at 180°C.

### Correlations Between Maillard Hazards and Physicochemical Properties of Air Fried French Fries

**Figure 2:**
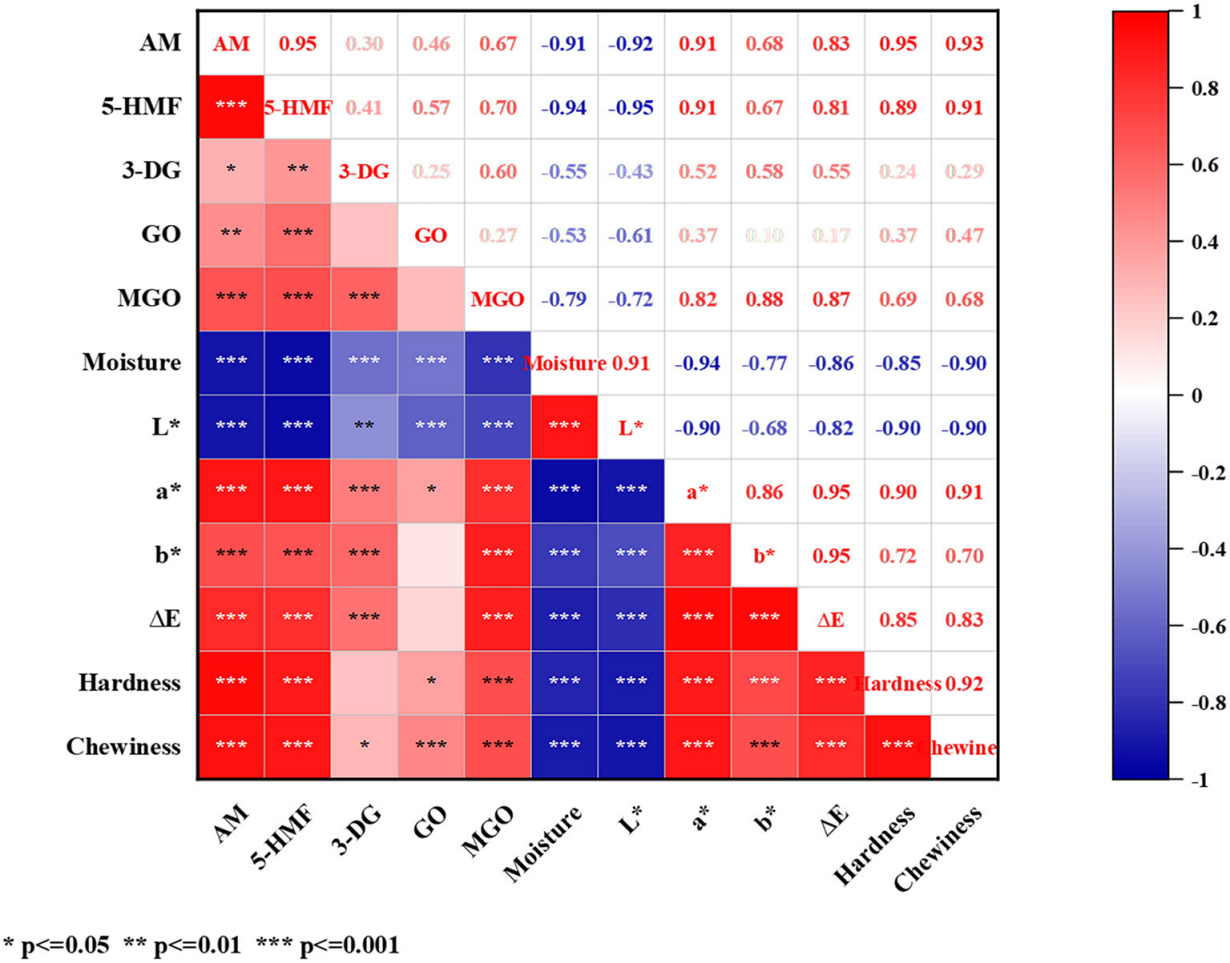
Heat map of correlation based on the concentration of AM, 5-HMF, αDCs, moisture content, color, and texture. Red and blue colors represented the correlation coefficients form 1 to −1. *p = 0.05; **p = 0.01; ***p = 0.001.

A heat map (Figure 2) was constructed to present the Pearson correlations between Maillard hazards (AM, 5-HMF, and αDCs) and physiochemical properties (moisture content, color, and texture). The contents of AM and 5-HMF were significantly (*p* < 0.001) negatively correlated with moisture and L^*^ value, and positively (*p* < 0.001) correlated with a^*^ and b^*^ value, ΔE, hardness and chewiness. For intermediates αDCs, the correlations between MGO and the physiochemical properties were similar to AM and 5-HMF, but the correlation coefficients between 3-DG/GO and the physiochemical properties comparatively lower.

### Starch Digestibility Characteristics of Air Fried French Fries

**Figure 3:**
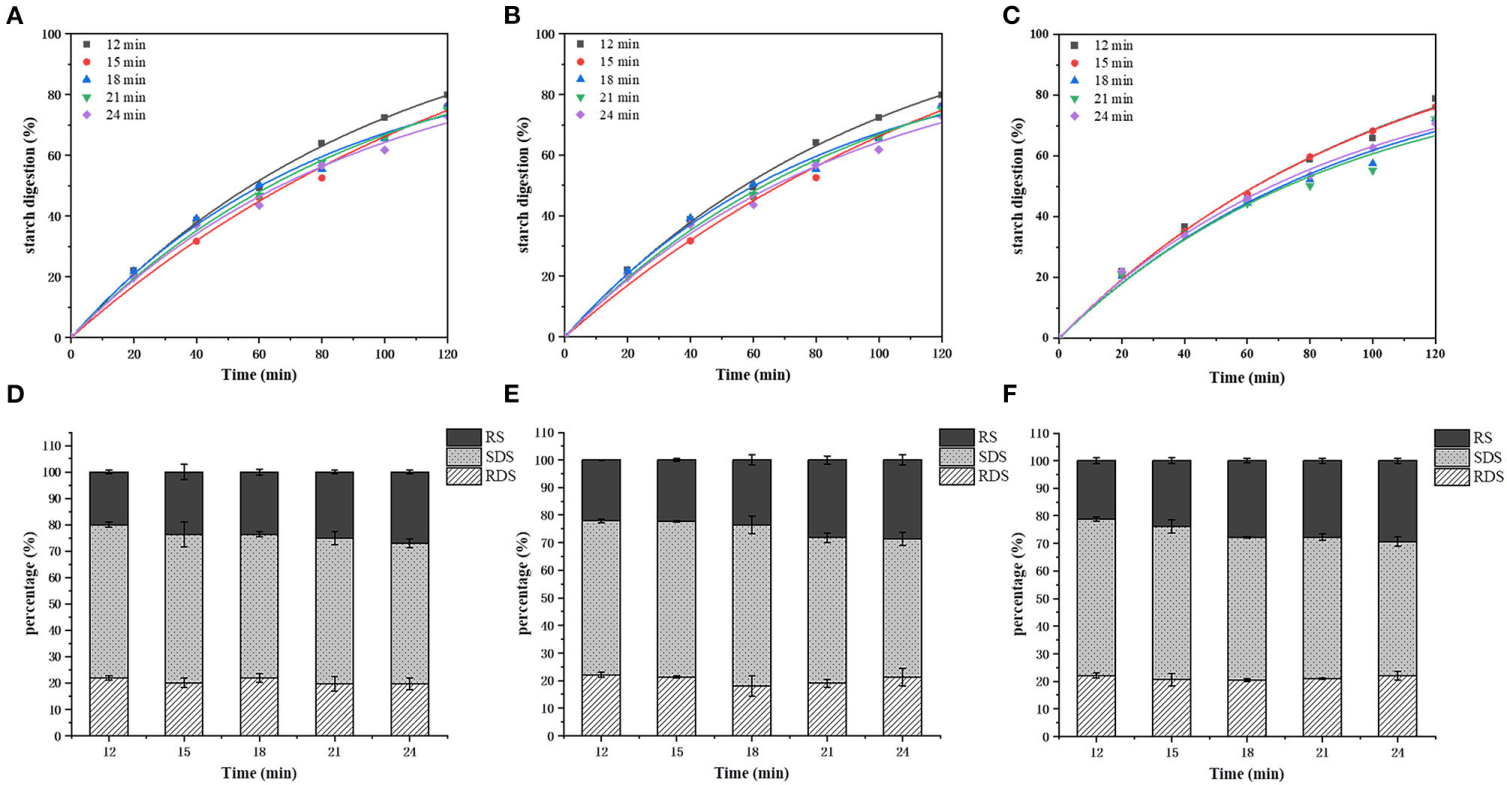
Changes of *in vitro* hydrolysis rate of air fried fries starch with air frying time at 180°C **(A)**, 190°C **(B)**, and 200°C **(C)**. The contents of RDS, SDS and RS in air fried fries under 180°C **(D)**, 190°C **(E)**, and 200°C **(F)** were changed with air frying time.

**Figure 4:**
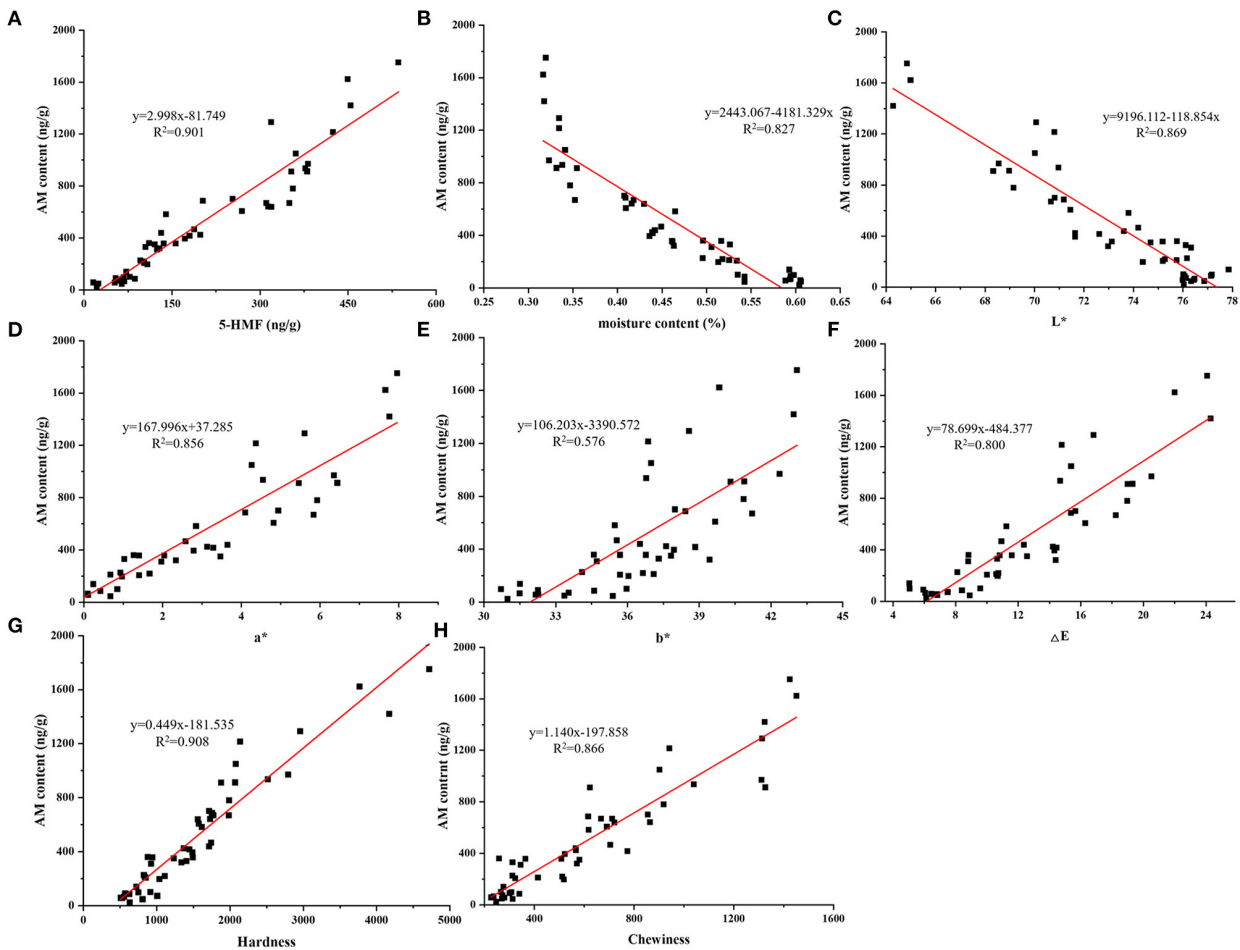
Linear regression lines between AM content and 5-HMF content **(A)**, moisture content **(B)**, L*(C), a*(D), b*(E), ΔE **(F)**, hardness **(G)**, and chewiness **(H)**.

The *in vitro* starch digestion results are shown in Figure 3. The *in vitro* digestibility of all samples increased as the enzymatic hydrolysis proceeded, and all the samples followed similar digestion trend but variations were obvious during the 40–120 min digestion. In general, the digestibility of the starch decreased with the air frying temperature and time (Figures 4A–C). The RDS, SDS and RS values of the air frying samples were 18.04% ± 3.63–22.12% ± 0.86%, 48.54% ± 1.74–58.42% ± 3.19%, 20.08% ± 0.65–29.34% ± 0.89%, respectively. No significant differences were observed for RDS among the treatments, SDS content decreased and RS content increased gradually with the air frying temperature and time.

### Maillard Hazards Content, Physicochemical Properties, Sensory Score, and Starch Digestibility of Deep Fried French Fried

The content of Maillard hazards, including AM, 5-HMF, MG, GO and 3-DG, physical characteristics, including moisture, texture, and color, and sensory score are shown in Table 3.

**Table 3:** The Maillard hazards content and physical characteristics of deep fried French fries.

**AM**	**HMF**	**MG**	**GO**	**3-DG**	**Moisture**	L*	a*	b*	**ΔE**	**Hardness/g**	**Chewiness**	**Sensory**
**(ng/g)**	**(ng/g)**	**(ng/g)**	**(ng/g)**	**(ng/g)**	**content (%)**							**score**
649.75 ± 16.86	314.73 ± 4.38	301.41 ± 5.79	231.76 ± 5.27	1164.90 ± 29.01	42.13% ± 0.61%	69.15 ± 0.57	2.17 ± 0.41	32.10 ± 0.93	13.39 ± 0.68	1687.89 ± 91.83	765.99 ± 69.67	98.5 ± 2.42

## Discussion

At sufficiently high temperatures can reduce some of hazards in food matrix and thus decrease the residues of it (35), but also accompany Maillard reaction, especially in carbohydrate-rich foods (such as fried potato products), resulting in the production of Maillard hazards (active intermediates αDCs, AM, 5-HMF, and polycyclic aromatic hydrocarbons) (36). The concentration of 3-DG was the highest among the αDCs compared to GO and MGO. 3-DG content showed a cumulative trend when air fired at 180°C, whereas 3-DG concentration firstly increased and subsequently declined when air frying at 190 and 200°C. This may be attributed to the promotion of the degradation of 3-DG with the increase of air frying temperature, making it more involved in the subsequent reactions (10). The correlation coefficients of MGO with 3-DG was 0.60 (*p* < 0.001), showing that MGO formed in air fried French fries may be mainly derived form 3-DG. This result is consistent with the previous study found that 3-DG can through the retroaldolization path to form MGO (37). There is no significantly correlation between 3-DG and GO, it may be due to that GO is mainly a product of autoxidation of glucose (38). A significant increase (*p* < 0.05) in AM formation as the air frying temperature and time increased, and the trend of 5-HMF was consistent with that of AM. AM was positively correlated with 5-HMF (*r* = 0.95, *p* < 0.01) of air fried French fries. This may be attributed to 5-HMF with a low melting point makes it thermodynamically favorable in the conversion of asparagine into acrylamide (39). Several studies have shown that αDCs were involved in the subsequent generation of AM and 5-HMF in thermal processed foods (10). The trend in the change of AM and 5-HMF content of air fried French fries was consistent with that of MG and GO with the time under the same air fried temperature. AM and 5-HMF were positively correlated with the three αDCs with correlation coefficient in the following order: MGO > GO >3-DG. αDCs can through the pathway of Strecker degradation to promote the formation of AM (10), and 80% of MGO as the precursor involved in the formation of AM in the Maillard reaction simulation system of glucose and asparagine (40). 3-DG can also be transferred into 5-HMF (10). The Maillard reaction is a series of chain reactions, the results in the present study and the previous reports proved that the three αDCs directly or indirectly participate in AM and 5-HMF formation.

Moisture content was certainly related to the formation of Maillard hazards (21). The moisture content of air fried French fries was decreased with the air firing temperature and time. We found that moisture content was negatively related to the content of αDCs, AM and 5-HMF of air fried French fries with the correlation coefficient order was 5-HMF (−0.94) > AM (−0.91) >MGO (−0.79) >3-DG (−0.55) > GO (−0.53). The reason of the correlation coefficient of αDCs was lower than AM and 5-HMF may be due to αDCs were the active intermediates of AM and 5-HMF, a certain of αDCs were consumed. The decrease of moisture content results in the increase in the activation energy of AM formation (21). This suggest that control the moisture content of foods can furtherly reduce the Maillard hazards formation. The accumulation of Maillard hazard is inseparable from the degree of Maillard reaction, which is visualized by the change in the color of the food surface. Color properties containing L^*^, a^*^, and b^*^ has been widely used to establish the correlations with the Maillard hazards in different foods (41, 42). According to the Pearson analysis, a negatively correlation (*p* < 0.01) between L^*^ value with the five Maillard hazards content, but a^*^, b^*^, and ΔE value was positively correlation (*p* < 0.001) with AM, 5-HMF, MGO, and 3-DG contents of air fried French fries. Correlation of L^*^, a^*^, b^*^ and ΔE value with AM and 5-HMF contents were higher than that of αDCs. The changes of the surface color of foods reflects the degree of Maillard reaction, which is closely related to the moisture content of food matrix. Therefore, the correlations of L^*^, a^*^, and b^*^ value with AM, 5-HMF content and moisture content were consistent. For texture, hardness and chewiness instrumentally indicate the sensory attributes during biting and chewing, and are often used to evaluate the quality of French fries. The textural properties of food are mainly affected by the spatial structure of food components and moisture (43). The trend in the change of hardness and chewiness were consistent with moisture content with the positively correlation coefficient was −0.85 and −0.90, respectively. Importantly, we found a significant correlation (*p* < 0.001) between moisture content and both Maillard hazards and physicochemical properties. Except for GO and 3-DG, the correlation coefficients are all >-0.77. This suggests that reducing the Maillard hazard exposure while maintaining the desired product quality can be achieved by controlling the moisture content of the air fried French fries.

Additionally, we evaluated the influence of air frying process on the *vitro* starch digestibility. More and more non-digestible polysaccharides in human gastrointestinal tracts are used in food processing to develop healthy foods such as low GI. They can be fermented by intestinal bacteria to generate short chain fatty acids that are beneficial to intestinal health (44). Potatoes contain a plenty of starch, and the effect of heat treatment on starch structure determines its GI. With the increase of air frying temperature and time, starch hydrolysis rate decreased and RS content increased. The temperature increase reduced the moisture content and increased the probability of the amylose molecules approaching each other in the aging recovery process, which was conducive to the formation of double helix structure and promoted the generation of RS. Air fried fries contain higher SDS content, which took long time for digestion, and was degraded to glucose after 120 min of enzymatic digestion. It has been proved that SDS does not cause a sudden increase of blood sugar, which benefits in balance blood sugar levels (27).

The deep fried fries and air fry fries were compared from the four aspects of Maillard hazards formation, starch digestibility, physicochemical properties and sensory scores to explore whether air frying can reduce Maillard hazards exposure while maintaining the expected product quality for the consumer. The optimal air frying process were 180°C−21 min, 190°C−18 min, and 200°C−18 min, with the sensory scores were 98 ± 2.58, 97.5 ± 2.64, and 97 ± 3.49, respectively, which were similar to sensory score of deep fried French fries (98.5 ± 2.42). Air fried samples had higher moisture content than deep fried fries. Deep frying is an immersive cooking process where a material remains in direct contact with hot oil at a temperature above the boiling point of water, which indicates greater heat and mass transfer, causing substantial water loss and high oil absorption (45), therefore, deep frying has lower moisture content. Nevertheless, due to lower heat and mass transfer, air frying process require longer cooking time and are often less crisp (20). Consumers are more satisfied with French fries with high lightness (L^*^), yellowness (b^*^) and lower redness (a^*^) (46). Compared with deep fried fries, the appropriately air fried fries with a longer heat treatment time, have higher L^*^, a^*^, and b^*^ values, characterized by higher lightness, higher brownness, and stronger golden yellow color. For texture, there was no significant difference in hardness compared with air fried fries and deep fried fries, but the chewiness was lower. Researches showed that an increase in water loss increased the hardness and chewiness of products (20). In terms of Maillard hazards content, 190°C−18 min showed the lowest production of AM (342.37 ng/g), which meant a reduction of 47.31% compared with deep frying (649.75 ng/g). Such a disparity in AM content between the two processes may be related to the factors such as oil content and oxygen. Previous reports confirmed that there was a significant (*p* < 0.01) correlation between oil content and AM formation (*p* < 0.01) (47). During deep frying process, AM can be formed not only through the Maillard reaction, but also from oil oxidation. Under high temperature conditions, the lipid oxidation promotes the formation of acrolein, and then acrolein further oxidized to acrylic acid, which can react with asparagine to generate appreciable amounts of AM (48), and the reduction rate of 5-HMF reached 57.04% at 190°C−18 min. In the model system, lipid oxidation products were reported to facilitate the formation of 5-HMF, then it can further generate AM, and some factors that promotes 5-HMF formation are therefore expected to promote AM formation (3). Moreover, there was no significant difference in RDS content between air fried and deep fried French fries, while air frying French fries had higher SDS content, lower RS content, and higher starch hydrolysis rate compared with deep-fried French fries. Due to the longer time required for air frying, a higher degree of gelatinization occurs, which makes it more easily available for enzymatic attack during digestion, thus increasing the starch hydrolysis rate (28). A dense crust was formed during deep frying that acted as a physical barrier to prevent digestive enzymes from penetrating into the starch granules and prevented starch from diffusing out of the disrupted starch granules, this may slow down starch hydrolysis in French fries (25). Studies have found that the RS content of French fries can be increased by adding fat or oil, ultimately reducing the process of starch digestion, thus it does not cause a sudden spike in blood sugar (27).

In this study, we clarified that Maillard hazards content increased with air frying temperature and time. Moisture content was significantly correlated with Maillard hazards content and physicochemical properties of air fried French fries, hence, reducing the Maillard hazard exposure while maintaining the desired product quality can be achieved by controlling the moisture content of the air fried French fries. Moreover, we found that although the physicochemical properties of optimal air fried and deep fried fries are slightly different, there is no difference in sensory evaluation; and the optimal air frying process can significantly reduce the formation of Maillard hazards; and air frying causes the starch digestibility change but couldn't cause a sudden spike in blood sugar. As a result, air frying can be used as an alternative process to fry potato strips, which is healthier and more attractive to customers.

## Data Availability Statement

The original contributions presented in the study are included in the article/supplementary material, further inquiries can be directed to the corresponding authors.

## Author Contributions

SW and H-nY: conceptualization and validation. LD and C-yQ: methodology, formal analysis, writing—original draft, and data curation. J-mL and JW: investigation and visualization. R-cW and YZ: writing—review and editing. All authors contributed to the article and approved the submitted version.

## Funding

This research was funded by the National Key R&D Program of China (2019YFC1606202).

## Conflict of Interest

H-nY was employed by MideaGroup Guangdong Midea Kitchen Appliances Manufacturing Co., Ltd. The remaining authors declare that the research was conducted in the absence of any commercial or financial relationships that could be construed as a potential conflict of interest.

## Publisher's Note

All claims expressed in this article are solely those of the authors and do not necessarily represent those of their affiliated organizations, or those of the publisher, the editors and the reviewers. Any product that may be evaluated in this article, or claim that may be made by its manufacturer, is not guaranteed or endorsed by the publisher.
